# The Genome Sequence of M228, a Chinese Isolate of Pseudomonas syringae pv. actinidiae, Illustrates Insertion Sequence Element Mobility

**DOI:** 10.1128/MRA.01427-18

**Published:** 2019-01-03

**Authors:** Joycelyn Ho, George Taiaroa, Margi I. Butler, Russell T. M. Poulter

**Affiliations:** a Department of Biochemistry, University of Otago, Dunedin, New Zealand; b Peter Doherty Institute, University of Melbourne, Melbourne, Australia; University of Maryland School of Medicine

## Abstract

We present here the complete genome sequence of M228, a Chinese biovar 3 strain of Pseudomonas syringae pv. actinidiae, a bacterial pathogen of kiwifruit. A comparison of the insertion sequence (IS) profile of M228 with that of ICMP18708, a New Zealand isolate of P. syringae pv. actinidiae, provided insight into the evolutionary history of IS elements within biovar 3.

## Abstract (precis)

We present here the complete genome sequence of M228, a Chinese biovar 3 strain of Pseudomonas syringae pv. actinidiae, a bacterial pathogen of kiwifruit.

## ANNOUNCEMENT

Pseudomonas syringae pv. actinidiae is the causal agent of a bacterial canker disease in kiwifruit (1). Strains of a globally distributed lineage are particularly virulent and have devastated orchards worldwide. This pandemic lineage is described as the hypervirulent clonal complex (CC HV) of biovar 3 (2). Pandemic isolates are very closely related (3, 4). The genome of M228, a nonpandemic biovar 3 strain, was sequenced and assembled. M228 is distantly related to the CC HV isolates and is grouped into CC China C (2).

M228 was isolated from a kiwifruit branch in 2010 in Shaanxi, China (5). After 72 h growth on King’s B agar at 26°C, DNA was isolated from M228 using the Mo Bio microbial DNA isolation kit (GeneWorks, New Zealand). The genome was sequenced using the PacBio RS II system and assembled with RS_HGAP Assembly.3 (Macrogen, South Korea). Subreads below the minimum length of 500 bp or with read quality of <0.80 were removed. The average length of the 142,738 filtered subreads was 9,576 bases, and the average depth of coverage was ∼200×. The Illumina sequencing library was prepared using the Illumina TruSeq DNA sample preparation version 2 kit following standard low-throughput protocols. Paired-end sequencing was performed (300- to 400-bp inserts, 80- to 100-bp reads) on a HiSeq 2000 system and demultiplexed using the Illumina Casava application, version 1.8.2. Primary reads were analyzed in FastQC (6), with low-quality reads removed and Illumina adaptors trimmed using Cleanadaptors (7). To identify errors in the HGAP assembly, a high-sensitivity read-mapping setting in Geneious 10.2.3 (Biomatters) was used to map 9,418,256 paired Illumina sequence reads to the initial assembly. The maximum allowed gaps and maximum mismatches per read were both set at 15%. For repeated sequences, multiple best matches were mapped randomly. The final assembly included a circular 6,674,594-bp chromosome and 72,748-bp plasmid, with GC contents of 58.3% and 56.2%, respectively. The genome of M228 differs from that of the New Zealand pandemic isolate ICMP18708 (GenBank accession numbers CP012179 and CP012180) by approximately one single-nucleotide polymorphism every 13,000 bp.

Bacterial insertion sequences (IS) are mobile elements that can replicate and integrate into new locations within a genome (8, 9). Although *de novo* insertions are frequent, deletions of IS are rare (10, 11). The repetitiveness of IS elements promotes intragenomic recombination, resulting in large-scale reorganization (8). The use of PacBio technology to generate the M228 genome sequence allowed a complete description of repetitive sequences, such as IS, which are difficult to identify and locate using short sequence reads.

**FIG 1:**
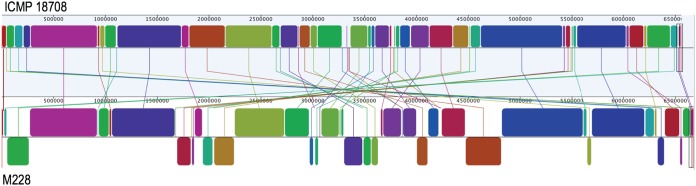
Mauve alignment (Geneious) (15) of the ICMP18708 (GenBank accession number CP012179) and M228 (CP032631) genomes, illustrating large-scale rearrangements.

A comparison of M228 with ICMP18708 revealed numerous internal recombination events (Fig. 1), with a subset being associated with IS. IS in M228 and ICMP18708 were located using ISsaga (http://issaga.biotoul.fr/ISsaga2), a semiautomatic annotation system (12). M228 carries 264 IS, while ICMP18708 carries 259. Of these, 210 IS were found at the same position in both strains. There were 54 unique IS locations in M228, whereas ICMP18708 had 49. The unique insertions included IS from seven different families (13). By analogy with other bacterial systems, it is probable that these unique IS locations represent insertions (8, 9). Methylation patterns from single-molecule real-time (SMRT) PacBio sequencing reveal a type I restriction-modification (RM) system recognizing GA^m^YCNNNNNCTGC (98.6% methylated) (14). This RM system is present at positions 6578 to 12939; the homologue present in ICMP18708 is disrupted by an ISPsy34 (13). The plasmids in M228 and ICMP18708 are highly similar, although both carry IS in unique positions.

### Data availability.

The M228 genome sequence has been deposited in DDBJ/ENA/GenBank under accession numbers CP032631 (chromosome) and CP032632 (plasmid). Sequencing reads were deposited in the Sequence Read Archive under accession numbers SRR8073200 (PacBio) and SRR8177059 (Illumina).
